# Navigating the challenges of NT-proBNP result disclosure in clinical research

**DOI:** 10.1017/cts.2025.10114

**Published:** 2025-07-25

**Authors:** Denise A. Kent, Michelle Villegas-Downs, Amanda Wilson, Jerry Krishnan, Lynn Gerald

**Affiliations:** 1 Department of Biobehavioral Nursing Science, Department of Medicine, University of Illinois Chicago, Chicago, IL, USA; 2 Department of Human Development Nursing Science, University of Illinois Chicago, Chicago, IL, USA; 3 Department of Community, Environment, and Policy, Mel And Enid Zuckerman College of Public Health, University of Arizona, Tucson, AZ, USA; 4 Breathe Chicago Center, Division of Pulmonary, Critical Care, Sleep, and Allergy, Department of Medicine, University of Illinois Chicago, Chicago, IL, USA

**Keywords:** Individual research Results, nt-probnp (N-terminal pro-B-type Natriuretic Peptide), clinical utility, research ethics, return of Results

## Abstract

**Background:**

The Office of Human Research Protections and the National Academy of Sciences, Engineering, and Medicine (NASEM) recommend the return of individual research results (IRRs) to study participants as a strategy to build public trust in science. However, the feasibility of sharing IRRs is unclear. Within a National Institutes of Health (NIH) funded parent study about Long COVID, we embedded the My ILLInet RECOVER Return of Results study to explore clinician-level considerations (e.g., validity, actionability, recommendations for follow-up) about returning a clinically used biomarker for heart failure (N-terminal pro-B-type natriuretic peptide, (NT-proBNP) collected as part of the NIH RECOVER study protocol.

**Approach:**

Clinicians participated in a three-phase modified Delphi process that sought their input to guide appropriate follow up recommendations the research team should provide to research participants with an abnormal NT-proBNP.

**Results:**

Clinicians agreed that NT-proBNP results could be returned to study participants. However, consensus was not reached on specific NT-proBNP thresholds that warrant immediate medical attention versus general follow-up.

**Discussion:**

Lack of clinical context presents a challenge in returning IRRs. Clinicians expressed concerns about the potential harm caused by misinformation or misinterpretation of these findings. While the NASEM report offers guidance on communicating IRRs, careful consideration is essential to ensure that clinical uncertainty is conveyed clearly, minimizing the risk of misinterpretation.

**Conclusion:**

The feasibility of returning IRRs to study participants depends, in part, on sufficient clinical context for the information to be actionable.

## Background

Individual Research Results (IRRs) are participant specific data generated during a research study [1]. IRRs differ from aggregate research data or study-level results which are shared at the end of the study with the scientific community (e.g., presentations at professional meetings, peer-reviewed abstracts, and publications) and to the general public (e.g., press release, public database ClinicalTrials.gov.) [2]. IRRs have the potential to benefit study participants and the broader research community. Reports from the Office of Human Research Protections (OHRP) and the National Academy of Sciences, Engineering, and Medicine (NASEM) suggest that IRRs can provide participants with valuable health related information [1,3]. Moreover, these reports indicate that returning IRRs may increase public engagement and trust in the research by improving the efficiency (e.g., speed of recruitment and rates of retention), generalizability, and participant-centeredness of research [1]. Developing thoughtful strategies for returning IRRs is critical for advancing ethical and impactful research practices.

There are ongoing debates about whether IRRs should be returned to participants. Supporters argue that research teams have an “ethical obligation” since that may provide participants with valuable information to maintain or improve their health [4 –6]. On the other hand, opponents highlight concerns about the uncertain value of IRRs outside of clinical context (e.g., symptoms, physical examination, medical history, medications, or prior test results) [7]. They caution that returning IRRs may cause unnecessary worry and could impose financial burden if participants seek medical care based on their results. Balancing the risks and benefits of returning IRRs is particularly challenging within clinical research [8]. Researchers must consider several critical questions related to returning IRRs, such as: (1) is the result valid, (2) is it actionable on its own, (3) does it require additional clinical context, and (4) what are the health implications of the result. Addressing these questions is essential to ensuring that the return of IRRs is both ethical and beneficial to participants.

To understand the complexities of returning IRRs to research participants, our team conducted a study (My ILLInet RECOVER Return of Results (MIRROR)) within the parent NIH-funded cohort study about Long COVID (Researching COVID to Enhance Recovery (RECOVER)) [9]. The MIRROR study had two aims: (1) solicit input from clinicians to inform our clinical center’s approach to return of IRR using N-terminal pro-B-type natriuretic peptide (NT-proBNP), a biomarker for heart failure, as an example, and (2) characterize the experiences, preferences, and expectations of research participants receiving IRR from the RECOVER study. The objective of this paper is to report findings from aim one. NT-proBNP was selected because dyspnea is a common symptom among individuals with Long COVID, and NT-pro BNP is often used clinically to differentiate cardiac from noncardiac causes of dyspnea [10 –12]. However, interpreting NT-proBNP levels is complex due to biological variability and demographic factors (e.g., age, gender, race) [13]. For example, research indicates that NT-proBNP levels tend to be higher in women, with a seven-fold higher likelihood of levels ≥125 pg/mL compared to men after adjusting for age, despite similar lifetime heart failure risks [14–16]. These variations highlight the challenges of interpreting NT-proBNP results in a research context. To address these challenges, our team consulted expert opinions from clinicians on whether and how NT-proBNP results should be returned to study participants.

## Approach

**Figure 1.**
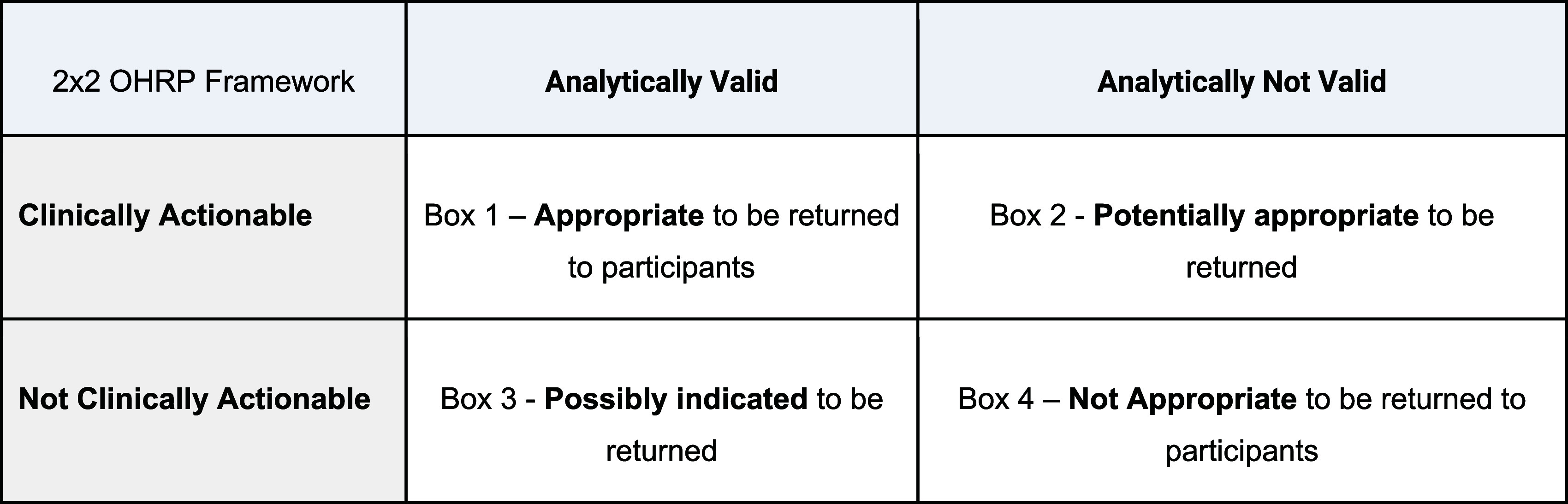
2x2 OHRP framework. Box 1 – individual results that are validated and actionable. These would include many clinical tests done in the context of research like pregnancy tests, liver function tests, EKGs, MRIs, and chest X-rays. There is a strong presumption of disclosure for these results. From a regulatory perspective, particularly in the medical field, “validated” will usually mean that the test or diagnosis is conducted using devices or assays that have regulatory approval from the food and drug administration (FDA) or certification under CLIA from the centers for medicare and medicaid services (CMS). Box 2 - results that are not validated but could be clinically actionable. An example is a genetic variant that is weakly associated with a heart condition, but the results have not been validated through a second study. The decision to return results in this category will be fact dependent. There is the possibility that the results in question becomes validated at a later date, either soon or long after the original study has concluded. Consideration should be given to providing results at that later date. Box 3 - results that are validated but not clinically actionable. An example here is a genetic diagnosis of huntington’s disease. This is a category where the concept of personal utility has gained traction. “Clinical utility” is usually from the physician’s perspective, but people might want and use information for their own purposes outside the clinical domain. Secretary’s advisory committee on human research protections (SACHRP) encourages disclosure in these situations, even though it may be contrary to current practice but consistent with the emerging set of responsibilities. Again, the decision to return results in this category will be fact dependent. There is the possibility that the results in question become clinically actionable at a later date, either soon or long after the original study has concluded. Consideration should be given to providing results at that later date. Box 4 - results that are neither validated nor clinically actionable. These can include experimental results in basic science studies, such as negative results when the subject might be found to have one biomarker or genetic variant or another that is not found to be associated with the disease of interest. There is not a strong presumption to make disclosure the default position for these types of purely experimental results and this is where the rebuttable presumption factors will have the most weight when considering them against the reasons for return.

**Figure 2.**
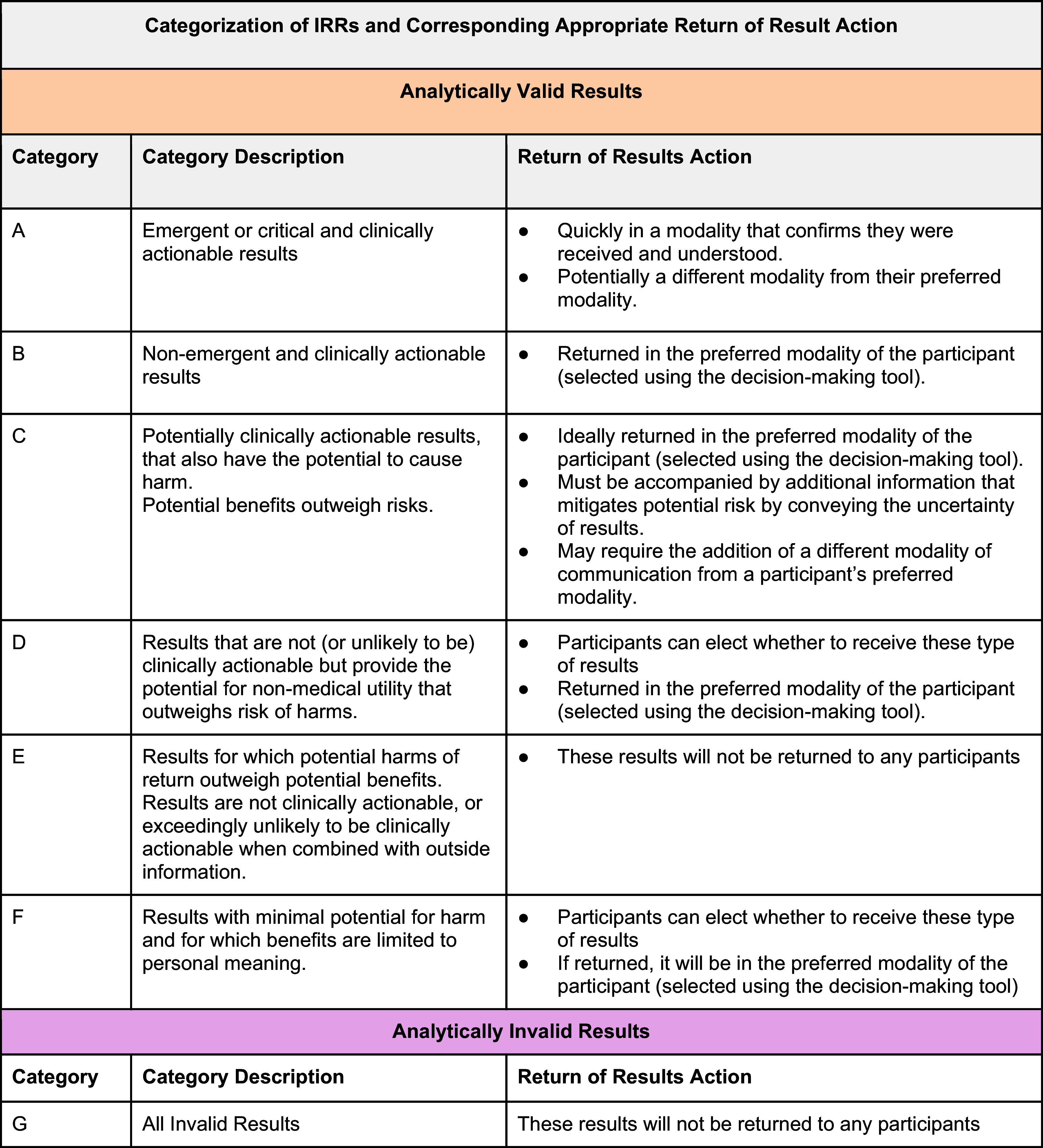
Categorization of IRRs and corresponding appropriate return action. Includes a table with seven categories for possible clinical actions. While our matrix is novel, it was developed using the guidance from the office of human research protections/Secretary’s advisory committee on human research protections which recommends returning results in-part based on their clinical utility which can be determined by categorizing those results in a two-by-two matrix of analytic validity and clinical actionability. We also used the 2018 National academy of science engineering and medicine report on return of individual research results which emphasizes the importance of considering the totality of benefits and risks related to the return of a particular result, which include clinical utility, but also, nonclinical (personal) utility, and personal meaning. (REF NASEM, OHRP, SACHRP).

A modified Delphi approach was used to guide the clinicians as they collaborated and discussed the return of NT-proBNP results to research participants [17,18]. To inform the decision-making process, we adapted two conceptual frameworks, see Figure 1 from the OOHRP and Figure 2 from the NASEM. These figures are rooted in the principles of clinical actionability and analytical validity and are crucial for decision-making in IRRs.

Figure 1 illustrates the OHRP’s 2x2 Framework that categorizes research results based on two dimensions: (1) Analytical Validity which refers to the ability of a test to accurately and reliably measure what it is intended to measure, and (2) Clinical Actionability which is the degree to which a result can guide medical or health decisions, typically involving established therapeutic interventions.

The framework includes four boxes:

Box 1. Valid and Actionable: Results are analytically valid and clinically actionable, such as many clinical tests (e.g., pregnancy tests, liver function tests). There is a strong presumption of disclosure for these results.

Box 2. Not Valid but Potentially Actionable: Results lack validation but could be clinically actionable (e.g., a genetic variant that is weakly associated with a heart condition). The decision to return these results is fact dependent.

Box 3. Valid but Not Actionable: Results are analytically valid but not clinically actionable, such as genetic diagnoses (e.g., Huntington’s Disease) for which no treatments are available. These may have personal utility.

Box 4. Neither Valid nor Actionable: Results that are neither analytically valid nor clinically actionable, (e.g., experimental results such as Xenon-129-MRI to assess lung structure and function). There is less presumption that these results must be disclosed.

Figure 2 builds on the OHRP framework and incorporates guidance from the NASEM report and the Secretary Advisory Committee on Human Research Protections. It categorizes IRRs into seven categories (A-G) based on their clinical utility and provides corresponding communication strategies for each category. The NASEM report emphasizes considering the totality of benefits and risks, including clinical utility, personal utility, and personal meaning when deciding whether to return results [1]. The development of these matrices reflects a novel contribution by integrating established frameworks with practical considerations for returning IRRs. They provide a structured approach to evaluating the return of results like NT-proBNP, ensuring that decisions are informed by both clinical actionability and analytical validity.

Convenience sampling was used to recruit three clinicians from different medical institutions who manage patients with NT-proBNP results in a clinical setting. The study was approved by the Institutional Review Board at the University of Illinois Chicago (UIC IRB #2022-1196). Clinicians were specifically selected based on their experience managing patients with normal and abnormal NT-proBNP levels in clinical practice, while clinicians involved in clinical research were excluded. This exclusion ensured the study focused on obtaining input grounded in practical, real-world experience rather than perspectives influenced by research-specific contexts. By targeting clinicians with direct experience interpreting and acting upon NT-proBNP results in clinical care, the study aimed to gather insights that would be most applicable to returning IRRs to participants [1,3].

Clinicians participated in a three-phase modified Delphi process that sought input to the following questions:

1. Is NT-proBNP clinically actionable when obtained as part of a research protocol?
2. What are the next steps that you would take after learning of your patient’s abnormal NT-proBNP?
3. Could an abnormal NT-proBNP drawn on a research participant ever trigger a duty to warn? If so, at what level would an abnormal NT-proBNP trigger a duty to warn?
4. Are there nonclinical benefits to returning NT-proBNP research results?
5. Are there participants for whom sharing NT-proBNP results would be harmful?
6. What is the appropriate follow up recommendation the research team should make for an abnormal NT-proBNP?

A 15-question electronic survey (phase 1) was emailed to clinicians and included detailed information on how IRRs were returned within the RECOVER study. Clinicians were then asked to provide evidence from the literature (e.g., clinical guidelines) to support their decision to return or not return NT-proBNP results to research participants. All survey responses were returned to the study team. The study team reviewed the clinicians’ responses for convergence or divergence in answers to the survey questions. Our team then hosted a Zoom meeting (phase 2) with the clinicians. The goal of the meeting was to clarify their responses and to see if consensus could be reached among the clinicians regarding the NT-proBNP result. Following the Zoom discussion, a second survey (phase 3) was sent to the clinicians to obtain final input on returning NT-proBNP results to participants.

## Results

Three clinicians were enrolled in the study: two physicians (one emergency medicine and one internal medicine hospitalist) and one acute care advanced practice nurse. Through the three-phase modified Delphi process, consensus was achieved that NT-proBNP research results should be returned to participants. Responses to specific questions are detailed below:

### Is NT-proBNP clinically actionable when drawn as part of a research protocol?

All three clinicians agreed that NT-proBNP results are clinically actionable even when obtained as part of a research study. They supported their decision to return these results based on published guidelines and peer-reviewed manuscripts [19–22]. The clinicians emphasized that, while NT-proBNP results can provide valuable clinical information for some participants, returning these results may also introduce diagnostic and therapeutic uncertainty for others. This uncertainty arises because the research setting often lacks comprehensive clinical information typically available in a clinical setting, such as symptoms, medical history, and concurrent treatments. These elements are crucial for accurately interpreting NT-proBNP levels and making informed treatment decisions. Consequently, the absence of detailed clinical context in a research setting complicates the interpretation of NT-proBNP results, leading to uncertainty about appropriate next steps.

### What are the steps you would take after learning of your patient’s abnormal NT-proBNP?

Clinicians agreed that having additional clinical information, such as chest X-ray, ECG, echocardiogram, renal function, and physical exam, would help guide their recommendation for follow-up. One clinician wrote, “….a full history (dyspnea on exertion, paroxysmal nocturnal dyspnea, orthopnea, leg swelling, abdominal swelling, nausea), and physical exam (pulmonary crackles, lower extremity swelling, jugular vein distention) to evaluate for clinical diagnosis of heart failure. I would also review the value of the abnormal NT-proBNP (slightly elevated vs. very elevated). I would also want to know the severity of their COVID-19 illness and their vaccination status.”

### Could an abnormal NT-proBNP result in a research participant ever trigger a duty to warn? If so, at what level would an abnormal NT-proBNP trigger a duty to warn?

All clinicians agreed that an abnormal NT-proBNP research result could trigger a duty to warn. However, there was no consensus among the clinicians about what specific value would prompt this warning. The clinicians suggested a wide range of thresholds, included: values >100 pg/mL, values >10,000 pg/mL, or indicated no single clinical threshold could be defined. One clinician wrote, “…a NT-proBNP < 900 pg/mL is the cutoff for lower limit. It’s difficult to find a study that shows at what level NT-proBNP is a critical value. There is one study that shows patients with NT-proBNP > 10,000 pg/mL on admission had a higher mortality so I used that value, but there is probably a lower value that would be similarly concerning (5,000 to 10,000 pg/mL)[23].” This lack of consensus highlights the need for individualized clinical judgment and further research to establish clear thresholds for action.

### Are there nonclinical benefits to returning NT-pro BNP individual research results?

When clinicians were asked if a NT-proBNP result obtained through research had any nonclinical benefits, only one clinician felt that the results could have personal utility or meaning to participants. The other two clinicians disagreed, stating a NT-proBNP value requires a provider interpretation within a clinical context and that abnormal results could potentially cause harm by leading to unnecessary worry. One of the clinicians stated, “yes, a BNP result may have both personal utility and personal meaning, and I would share the result with the person and advise them to seek further follow up with their PCP.”

### Are there participants for whom sharing NT-proBNP results may be harmful?

Two of the three clinicians noted that giving participants a NT-proBNP result without additional clinical context could cause a person to worry and prompt them to search online for answers. As one clinician explained, “it may cause emotional distress to the patient if they start to “google” the cause for the elevation/abnormality. For example, they may start to think they have acute heart failure or an increased risk of morbidity or mortality.” The third clinician felt that sharing the result would cause little or no harm unless the person already had untreated anxiety. However, this clinician also cautioned that harm could occur if other practitioners only responded to the lab value alone which could lead to unnecessary testing and treatment.

### What is the appropriate follow up recommendation the research team should make for an abnormal NT-proBNP?

Clinician responses varied regarding appropriate follow-up for an abnormal NT-proBNP research result. One clinician emphasized that the presence of symptoms should guide whether follow-up occurs in urgent care, emergency department, or with a primary care provider. Another clinician suggested that NT-proBNP value level between 900–10,000 pg/mL could be managed by a primary care physician in a nonurgent setting. The third clinician indicated that the primary care provider is generally the best starting point for follow-up unless the participant shows signs of acute decompensated heart failure, in which urgent evaluation and treatment in an emergency department is necessary.

## Discussion

The results from our modified Delphi process illustrate the challenges research teams face when returning IRRs such as, NT-proBNP to participants [24–26]. The clinicians agreed that NT-proBNP results obtained through research are clinically actionable and should be returned to participants. However, clinicians did not reach consensus on specific thresholds that would trigger immediate medical attention versus general follow-up. Additionally clinical context was needed to fully interpret these results.

Using the Categorization of IRRs table (Figure 2), clinicians evaluated the overall benefits and risks associated with returning NT-proBNP results. The clinicians emphasized the importance of clear communication to avoid misinterpretation and ensure that participants understand their results. Figure 2 also helped clinicians develop tailored communication strategies.

Despite the agreement that NT-proBNP results were both clinically actionable and analytically valid, clinicians did not come to a consensus on whether a specific NT-proBNP value would indicate emergent and clinically actionable versus nonemergent and nonclinically actionable results. This process revealed several important considerations and challenges. First, NT-pro BNP results are generally considered clinically actionable but requires careful consideration within the individual’s clinical context. Secondly, there is no consensus on the specific NT-proBNP levels that would trigger a duty to warn, emphasizing the need for case-by-case evaluation. Finally, the potential for both benefits and harms in returning NT-proBNP results underscores the importance of thoughtful communication and follow-up strategies.

The application of Figures 1 and 2 is a novel approach to determine what results should be returned to participants and what supporting clinical information (e.g., participant-centric education) should accompany them. Both figures emphasize the importance of clinical actionability and analytical validity in determining whether IRRs should be returned. Clinicians did not provide specific feedback on the utility of Figures 1 and 2 but acknowledged their importance as structured tools for interpreting and communicating IRRs. To gather direct feedback on the utility of these tools, future studies could include specific questions in surveys or discussions to assess how clinicians perceive the effectiveness and practicality of these tools for decision-making. This would help refine the tools to better meet the needs of clinicians and researchers involved in returning IRRs.

All clinicians discussed concerns about potential harms from misinformation or misunderstanding of research results, a concern also reflected in the literature [27 –30]. Therefore, study teams should carefully evaluate the clinical relevance, analytical validity, and potential impact of returning research results to participants. Clear communication strategies are essential to ensure that results are accurately interpreted and do not lead to misinformation or unnecessary concern. Additionally, ethical considerations – such as participant autonomy, privacy, and the duty to warn – must guide decisions on returning IRRs. The broader implications of this work extend beyond individual studies, influencing participant trust, engagement, and the future of research. Establishing best practices for returning IRRs can enhance public confidence in scientific research, improve recruitment and retention rates, and ultimately contribute to a more participant-centered research environment.

One limitation of this study was the small sample size of clinicians involved in the Delphi panel (*n* = 3). Despite its small scale, this study serves as a model for a large clinician pool to provide insights into interpreting and communicating IRRs in a research setting. Additionally, research should focus on developing implementation strategies that address clinical uncertainty while ensuring that participants receive clear, concise messages about their results. Aligning these strategies with existing guidance from organizations such as NASEM would further support ethical and responsible practices in returning IRRs. By addressing these considerations, study teams will be better equipped to navigate the complexities of returning IRRs.

## Conclusion

This study contributes to the science of return of IRRs in two ways: (1) demonstrates the utility of a modified Delphi approach for structuring discussions to determine if and when research results should be returned to participants, (2) emphasizes the importance of clinical context to support clinician interpretation. This study further highlights the need for comprehensive guidelines and tools to support research teams in returning IRRs to participants. Such resources should address the nuances of result interpretation and communication strategies to support participants sharing their research results with their clinician for contextualization and for appropriate follow-up recommendations. Future research should focus on developing guidelines and implementation strategies for returning results to participants, particularly in cases of clinical uncertainty. The return of NT-proBNP research results to participants requires a balanced approach that considers clinical actionability, potential benefits and harms, and the need for clear communication. As the field of returning IRRs continues to evolve, studies like this one contribute valuable insights to inform best practices and improve participant outcomes in clinical research.

## Data Availability

All data and materials are available upon request. Please send requests to the corresponding author.
